# Linear Dependence of Sublimation Enthalpy on Young’s Elastic Modulus: Implications for Thermodynamics of Solids

**DOI:** 10.3390/ma18153535

**Published:** 2025-07-28

**Authors:** Anne M. Hofmeister

**Affiliations:** Department of Earth, Environmental, and Planetary Sciences, Washington University, St. Louis, MO 63130, USA; hofmeist@wustl.edu

**Keywords:** sublimation, elastic moduli, rigidity, enthalpy, 2nd law of thermodynamics, interatomic forces, molecular solids, metals, energy reservoirs, structure

## Abstract

Classical thermodynamics omits rigidity, which property distinguishes solids from gases and liquids. By accounting for rigidity (i.e., Young’s elastic modulus, ϒ), we recently amended historical formulae and moreover linked heat capacity, thermal expansivity, and ϒ. Further exploration is motivation by the importance of classical thermodynamics to various applied sciences. Based on heat performing work, we show here, theoretically, that density times sublimation enthalpy divided by the molar mass (*ρ*Δ*H*_sub_/*M*, energy per volume), depends linearly on ϒ (1 GPa = 10^9^ J m^−3^). Data on diverse metals, non-metallic elements, chalcogenides, simple oxides, alkali halides, and fluorides with cubic structures validate this relationship at ambient conditions. Furthermore, data on hcp metals and molecular solids show that *ρ*Δ*H*_sub_/*M* is proportional to ϒ for anisotropic materials. Proportionality constants vary only from 0.1 to 0.7 among these different material types (>100 substances), which shows that the elastic energy reservoir of solids is large. Proportionality constants depend on whether molecules or atoms are sublimated and are somewhat affected by structure. We show that ductility of refractory, high-ϒ metals affect high-temperature determinations of their Δ*H*_sub_. Our results provide information on sublimation processes and subsequent gas phase reactions, while showing that elasticity of solids is the key parameter needed to assessing their energetics. Implications are highlighted.

## 1. Introduction

Currently popular microscopic models of heat storage and heat flow in solids are based on historical thermodynamic treatments of the behavior of gases. As discussed previously [1], this analogy is undermined by the extremely different behaviors that characterize these two states of matter. Dichotomies are as follows:

1. Solids alone are rigid, thereby elastically resisting shear forces, and propagating shear waves;
2. Diffusion of heat and mass in a gas are inseparable consequences of molecular translation, whereas in solids these processes are entirely independent;
3. Heat in monatomic gases is stored in long-range atomic translations, but atomic motions in monatomic solids minimally perturb their positions in the structure: Thus, vibrational energy (stored heat) must be smaller than the energy reservoir associated with maintaining the lattice configuration of monatomic solids;
4. Similarly, the lattice energy reservoir for a more chemically complex solid exceeds the energy associated with local translations, whereas the main energy reservoir of molecules in gases are in their long range translations. Both complex solids and chemically analogous molecular gases have additional vibrational energy stored in local modes which are restricted in the solids, and thus have lower energy than its elastic reservoir.

The elastic behavior of a solid is foundational to continuum mechanics [2], but this large energy reservoir is not part of classical thermodynamics.

Omission of rigidity from discussions of solids effectively neglects the 2nd law of thermodynamics. Specifically, adding heat to a body not only raises its temperature, but moreover performs pressure–volume (*P*–*V*) work to expand the body as it warms. Internal expansion of a solid is opposed by its rigidity [1]. In contrast, liquids and gases deform (flow) under any stress [3]. Moreover, the response of liquids (viscous drag) is inelastic [4], which response in solids is in addition to elastic (reversible) changes. In contrast, gases offer negligible resistance, elastic or inelastic.

Based on elastic solids resisting expansion during heating, Hofmeister et al. [1] derived new formulae involving specific heat (*c_P_*, J g^−1^), by assuming a perfectly frictionless response. For example, volumetric thermal expansivity (α) is described by:

(1) $$\alpha_{\mathrm{vol}}\approx \frac{\rho c_{P}}{\Upsilon },\;\mathrm{where}\;\alpha_{\mathrm{vol}}\equiv \frac{1}{V}{\left.\frac{\partial V}{\partial T}\right|}_{P}=\frac{1}{\rho }{\left.\frac{\partial \rho }{\partial T}\right|}_{P},$$

where *ρ* = density and ϒ = Young’s modulus (i.e., rigidity: Section 2 provides details), *V* is volume, *T* is temperature, and *P* is pressure. The conventional symbol *E* is not used here because *E* represents internal energy in thermodynamics, and is elsewhere commonly used to represent energy. The units of ϒ are pressure. As 1 GPa is equivalent to 10^9^ J m^−3^, Young’s modulus describes energy density.

The left hand side (LHS) of Equation (1) originates in ϒ representing an average force per area about an average atom in any given solid, while assuming spherical symmetry. Equation (1) was experimentally validated, allowing for geometric departures from spherical symmetry, for diverse metals and simple insulators at ambient conditions and for 10 of these solids over wide temperature ranges [1]. Section 2.2 and Appendix A provide details relevant to the present report.

The results of any such macroscopic approach are independent of the microscopic mechanism(s) involved. Thus, our model [1] is independent of possible roles of phonons, as opposed to participation of electrons or photons, in heat storage and/or heat transport. Furthermore, by recognizing that heat stored in the solid is distinct from the heat that is flowing through the solid (Point 2, above), a subsequent paper [5] explained why experimental values for thermal diffusivity (*D*) and *c_P_* change in the opposite directions at phase transitions and commonly with increasing *T*. Findings of [1,5] motivated the present study.

### 1.1. Purpose of the Paper

To further explore the role of rigidity in thermodynamic assessments, which pertains to both internal forces and energetics of solids, the present paper considers sublimation. During this process, the constituents of the solid are dispersed into a gas; hence, rigidity is fully lost. As in previous work [1,5], an ideal, frictionless elastic regime is considered. This state is denoted PFES for Perfectly Frictionless Elastic Solid (discussed further below). Glasses (amorphous solids) are not investigated since they partly respond to stress via viscous deformation. Likewise, melting is not covered because melting interchanges rigidity with viscosity. Although gases have viscosity, the process is predominantly diffusion of momentum which promotes flow, rather than impeding flow: See, e.g., discussion of the kinetic theory of gas in [6] (pp. 143–179).

We focus on cubic structures to minimize complicating effects of anisotropy, and on ambient conditions, where most measurements of ϒ are made. Diverse types of crystalline solids are explored (e.g., metals vs. semi-conductors vs. insulators; monatomics vs. diatomics vs. polyatomics; weak vs. strong materials) to elucidate effects of structure and chemical bond-types on sublimation. Molecular solids are investigated to probe effects of significant anisotropy and spontaneous sublimation at low *T*.

### 1.2. Organization and Outcomes of the Paper

Sublimation is complicated because surfaces are involved (e.g., [7]) and high *T* experimental conditions can promote secondary reactions in the gas. However, classical thermodynamics represents the heat of sublimation as a single numerical value irrespective of sample size, i.e., bulk material is represented. This macroscopic approach needs discussion. Also, continuum mechanics may not be familiar to those applying classical thermodynamics. Hence, Section 2 provides background material on sublimation and rigidity, in addition to summarizing our recently developed theory of the energetics of solids. Section 3 describes our methodology. Section 4 compares enthalpies of sublimation (Δ*H*_sub_) to elastic moduli of solids, primarily to *Y*. The roles of structure, chemical bond type, density, molecular weight (*M*), and compressive vs. shear moduli are explored. We find that energy density (*ρ*Δ*H*_sub_/*M*) depends directly and simply on ϒ. Section 5 extracts information on the process of sublimation process from our results and discusses implications of our findings and future work. Section 6 concludes.

## 2. Theoretical Basis and Background

In the materials science and engineering literature, ‘elastic’ denotes materials that return to their original shape after deformation, where some heat generation likely accompanies applying stress [8]. Low stress is implied because above some finite applied stress, a solid will permanently deform even when temperatures are below melting (e.g., plasticity, see [2]). Our thermodynamic analysis uses the original definition of ‘elastic’ from physics, which denotes conservation of mechanical energy. Considering ‘perfectly frictionless elastic solids’ (PFES) here is consistent with assuming reversibility and comparing states while neglecting processes, which describes classical thermodynamics. Section 2.1 and Section 2.2 describe elastic properties, our previous model [1], and its extension to sublimation (Section 2.2.2).

Sublimation is a process, which in thermodynamic assessments, is treated as a transition. Section 2.3 discusses limitations of this assumption by summarizing measurements of sublimation where species in the gas produced are quantified.

### 2.1. Definitions and Behavior of Elastic Moduli

In the elastic regime, the response of a cubic solid is commonly described by:

(2) $$B_{\mathrm{aco}}=\frac{volumetric\;stress}{volumetric\;strain};\;G=\frac{shear\;stress}{stear\;strain},$$

Shear moduli (*G*) are determined via elasticity studies which also yield bulk moduli (*B*). Commonly used methods involve acoustic (subscript aco) waves, ultrasonic pulses [9], or stimulation by light (Brillouin spectroscopy [10]).

Additionally, bulk moduli are independently extracted by measuring contraction during compression:

(3) $$\frac{1}{B_{T}}\equiv -\frac{1}{V}{\left.\frac{\partial V}{\partial P}\right|}_{T}=\;\frac{1}{\rho }{\left.\frac{\partial \rho }{\partial P}\right|}_{T}\;.\;$$

Within uncertainty, compression experiments provide the same *B* as in elastic studies for hundreds of well-studied materials at ambient conditions and a smaller subset at elevated temperature [1]. Equivalence exists for four distinct reasons:

- Frictional heat is generated by the apparatus in compression experiments, whereas pulses in elastic experiments energize vibrations. The latter occurs since acoustic modes are tied to the heat capacity and thus to heat storage in solids (e.g., Debye’s historical model, e.g., [11]). Heat always flows, per Fourier’s laws. Because little heat is added (and because this small augmentation is diffused away over the duration of the measurement) in either experiment type, both are approximately isothermal, providing the equivalence shown by [1].
- Under reversible conditions, entropy (*S*) is defined as *Q*/*T*, where *Q* is heat. Elasticity studies are denoted as adiabatic (constant *Q*), considered to be reversible, and labeled as isentropic (constant *S*), which requires *T* to be constant. See [6] (pp. 8–17) for additional discussion.
- The chain rule for derivatives points to only one bulk modulus existing [1]. This stems from mass and heat independently occupying any given space in classical physics and is evident in the definition of α in Equation (1), right hand side (RHS), where *V* depends on *T*, and that expansion while holding *S* or *Q* constant is not considered in classical thermodynamics.,
- Importantly, we add here that the elastic definition of *B* (Equation (2)) and its equation of state definition (Equation (3)) are both hydrostatic, since only the change in volume is germane. Hydrostatic conditions are unique in that these require uniform, non-directional forces and stresses.

In compression experiments, maintaining hydrostatic conditions is an important goal.

Elasticity is alternatively represented by Young’s modulus and Poisson’s ratio (*ν*):

(4) $$\Upsilon =\frac{longitudinal\;stress}{longitudinal\;strain};\nu =\frac{lateral(transverse)\;stress}{longitudinal\;strain}.$$

Young’s modulus measures a material’s resistance while distorting and contracting under a mechanical load, i.e., its rigidity. Poisson’s ratio compares a material’s resistance to distortion under a mechanical load rather than the material’s propensity to alter in volume during distortion [12]. All stable isotropic materials have ν between −1 and + ½. Whereas *ν* is dimensionless, pressure is the unit describing *B*, *G*, and ϒ.

#### 2.1.1. Why Elastic Moduli Affect Sublimation

Young’s modulus was used to describe the resistance of the solid to incremental expansion by [1], after Orowan’s use of ϒ to represent tensile strength [2]. Thus, Young’s modulus pertains to sublimation, where a solid expands to provide a gas. Resistance of a solid is only partially governed by the bulk modulus, since solids also shear: see Meyers and Chawla [2] for discussion of Frenkel’s theory for shear strength.

The link to sublimation (detailed below) is simple because ϒ = 0 for a gas, as is evidenced by the relationship between elastic properties for isotropic materials, which case includes gases:

(5) $$\Upsilon =\frac{9BG}{3B+G}=2G\left(1+\nu \right)=3B\left(1-2\nu \right)\;\mathrm{and}\;\nu =\frac{3B-2G}{6B+2G}.$$

The second most important modulus during sublimation should be *B* since dispersal involves a large increase in volume. This tie is also evident in Equation (5) since *B* of most materials (but not diamond) is significantly larger than *G*. Gases resist volumetric compression. For the ideal gas *B* = *P*, and so *B* of evolved gas is negligibly small at 1 atm conditions investigated here.

#### 2.1.2. Directionality and Elasticity

Equation (4) implies that *Y* depends on direction. Hence, we focus on isotropic solids, which structures are the same in every direction. The elasticity matrix, a 2nd order tensor (e.g., [2] (p. 96)), simplifies to three stiffness coefficients for isotropic solids: *c*_11_, *c*_44_, and the off diagonal element *c*_12_. These three coefficients combine to yield the two elastic moduli:

(6) $$B=\frac{\left(c_{11}+2c_{12}\right)}{3}\;\mathrm{and}\;G=\frac{1}{2}\left[c_{44}+\frac{\left(c_{11}-c_{12}\right)}{2}\right].$$

Two moduli (Equations (5) and (6)) suffice for isotropic solids because there are only two types of elastic waves: In the longitudinal type, particles move in the direction of propagation whereas in the transverse type of waves, particles move in the plane normal to the direction of propagation, e.g., [13] (pp. 103–122). Although there are two transverse directions in 3-dimensional space, cubic (isotropic) symmetry renders these identical, thus reducing the parameters needed from three to two. The longitudinal wave differs from transverse because applying elastic waves to a material perturbs its atomic arrangement, effectively reducing its symmetry during flow.

For anisotropic solids, Hill’s average is used to determine elastic moduli from stiffness coefficients. Hill’s average (see, e.g., [14]) represents the equivalent isotropic polycrystalline aggregate. Hill averaged Voight’s and Reuss’ approximate averages. These formulae depend on symmetry and can be quite complex [15], and so are not reported here for the few anisotropic solids considered.

#### 2.1.3. Relationships of Elastic Moduli for Metals and Diatomics

**Figure 1:**
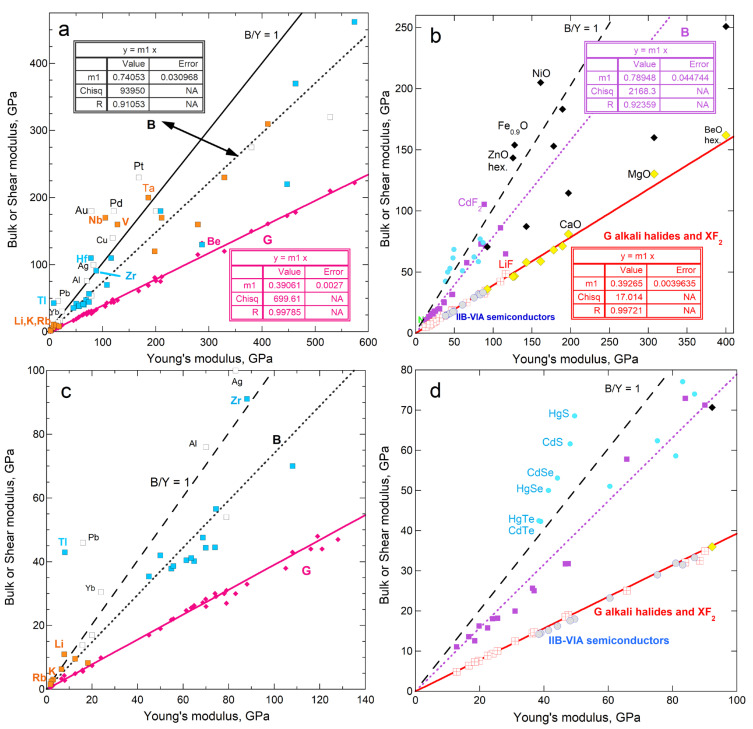
Dependence of bulk and shear moduli on Young’s moduli for materials investigated in the present paper. Least squares fits are shown (NA = not applicable). Samples with *B* > ϒ are labeled: (**a**) Metallic elements with fcc, bcc, and hcp structures. Pink diamonds and left y-axis provide *G*, where Be is the only metal departing significantly from the trend. Open squares and right y-axis show *B*. Samples with *B* > ϒ are labeled, where orange interiors = bcc metals; blue = hcp metals; and white = fcc metals; (**b**) Cubic diatomic solids, plus hexagonal BeO and ZnO for comparison. Other labeled points indicate samples with *B* > ϒ. Fits are only to the alkali halides and fluorite structure compounds (red squares with cross = *G*; purple squares = *B*). Projections of the lines describe the chalcogenides (blue circles with gray centers = *G*; aqua circles = *B*) and the oxides (diamonds with yellow centers = *G*; black diamonds = *B*); (**c**) Expanded view of the metals; (**d**) Expanded view of the diatomics. Data are publicly available [16,17] in compilations, where reported uncertainties vary, but are generally 1 to 2%, i.e., roughly symbol size. Section 3 and Section 4 give further information.

Regarding cubic solids with simple chemical compositions and close-packed hcp metals, Figure 1 shows that *G* and ϒ are linearly related. To the best of our knowledge, this correlation has not been previously published.

The solids used to construct Figure 1 are considered in evaluating the connection of sublimation and rigidity (Section 4). Implications of the correlation in Figure 1 for elasticity are beyond the scope of the current report and are not discussed here. However, the close match of *G* with 0.39 ϒ, while *B* is scattered about ~0.75 ϒ, pertain to sublimation energy, and are discussed in Section 5.

### 2.2. Implications of Pressure–Volume Work Being Performed During Addition of Heat

Our steady-state model [1] considers perfectly elastic changes in a frictionless solid (PFES) upon receipt of additional heat, which also raises its temperature. As is typical of classical thermodynamic assessments, we assume:

- Changes are reversible, which is possible for a perfectly elastic frictionless solid;
- Heat is added incrementally, thus maintaining steady-state, which reasonably describes the experiments;
- Mechanical energy is conserved, which permits separate treatment of variables related to mass occupying space (i.e., thermal expansivity and the elastic moduli) from behavior of heat occupying space (i.e., heat content *Q* and specific heat).

#### 2.2.1. Role of Specific Heat in Thermal Expansion

During measurements of thermal expansivity, pressure is held constant while adding heat to the system raises the temperature over some finite time via diffusion. This increase in *T* causes *V* to increase in accord with Equation (1) (RHS).

Time affecting expansion is accounted for in dilatometry experiments by adjusting the heating rate and using standards with thermal diffusivity similar to that of the sample [18]. In crystallographic experiments, measurements are at intervals and are slow, so heat diffusion has a negligible impact.

For an ideal PFES, external addition of heat performs *P*–*V* work:

(7) $$dQ_{\mathrm{ext}}=work=PdV.$$

Experiments do not measure stored *Q* directly, but rather record the response of matter to augmentation of heat. Conserving mass makes specific heat germane, which is defined in terms of the heat externally supplied in order to raise a unit mass of some material by one degree:

(8) $$c_{P}\equiv \frac{1}{M}{\left.\frac{\Delta Q_{\mathrm{ext}}}{\Delta T}\right|}_{P}={\left.\frac{1}{\rho V}\frac{\partial Q_{\mathrm{ext}}}{\partial T}\right|}_{P}\;.$$

Heat capacity is similar to specific heat (*c_P_*) but is computed on a per-mole basis, rather than per mass.

Combining the definitions of Equations (7) and (8) gives [1]:

(9) $$\;c_{P}M\Delta T=\Delta Q_{\mathrm{ext}}=work=PdV=F\Delta L,$$

where *F* is the force needed to expand the bond with length *L*. Assuming that *F* = ϒ × area, and that the volume about an atom is spherical [1], gives:

(10) $$c_{P}M\approx \Upsilon 4\pi L^{2}\frac{\Delta L}{\Delta T}=\Upsilon 4\pi L^{3}\frac{\Delta L}{L\Delta T}=\Upsilon V\alpha_{\mathrm{vol}}.$$

Rearranging terms and using *V* = *M*/*ρ* provides Equation (1) (LHS).

However, atoms in solids are not subject to a perfectly spherical force field. Hence, Equations (1) (LHS) and (10) do not account for solids having a variety of structures with different bonding arrangements and a variety of forces around the constituent atoms. Generalizing and re-arranging Equation (10) provides:

(11) ρcPαvol=constant #1 ×Υ,

where the proportionality constant depends on the number of bonds around an atom, i.e., on the crystallographic structure. Chemical bond type (i.e., the electronic configuration) should also have an effect.

**Figure A1:**
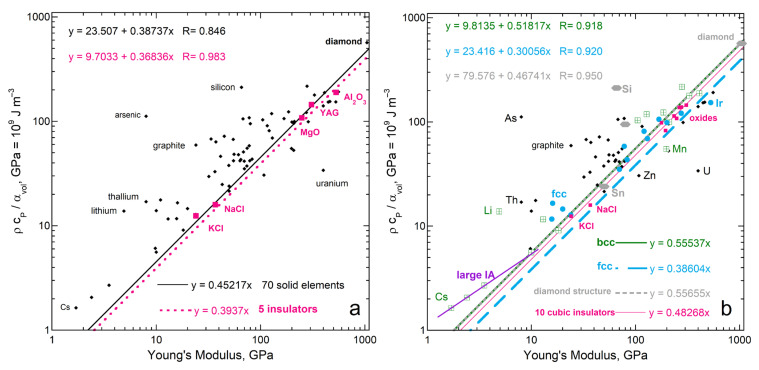
Evaluation of Equation (11), via least squares fits. The trends shown assume no intercept. For comparison fits with an intercept are listed to the upper left, which provide residuals: (**a**) Behavior of all solid elements (black diamonds), using data from [16] and data on the 5 simplest chemical compositions of insulators (pink squares) compiled by [50]; (**b**) Same plot, but the elements with cubic structures are distinguished from the rest. Blue dots = fcc; Green square with cross = bcc; Gray polygon = the diamond structure. Purple line = a power law fit to alkali metals, excluding Li, *ρc_P_*/α = 1.1291ϒ^0.6937^, with a residual of 0.99. The 9 insulators from [50] are NaCl, KCl, MgO, CaO, MnO, spinel (Mg_2_AlO_4_) Yttrium Aluminum Garnet (YAG), and two alumino-silicate garnets (Mg-rich and Ca-rich). Non-cubic Al_2_O_3_ is omitted. Modified after work by the author [1], which work has a Creative Commons License.

Simple structures were considered in [1] to estimate constant #1 from geometry alone. Appendix A reproduces salient results from this study. From Figure A1:

- The proportionality constant of Equation (11) depends primarily on the crystallographic structure of the solid;
- Cubic structures are well-represented by Equation (11);
- Constant #1 is on the order of ½ for several different cubic structures.

#### 2.2.2. Derivation of Work Performed During Sublimation

Dispersing the solid requires adding heat, which performs the work needed to disperse the solid per the 2nd law. Previous analyses of this process consider that the volume of the solid is held constant. Work performed is thus described by:

(12) $$\;\Delta Q_{\mathrm{ext}}=c_{V}M\Delta T=work=V\Delta P$$

Using Δ in Equation (12) signifies that states are being compared, as in classical thermodynamics.

Sublimation is generally evaluated thermodynamically without specifying *T* changes (Section 2.3), and so specific heat is not germane. The heat added on a per-mole basis is the enthalpy of sublimation (Δ*H*_sub_, typically reported in kJ mol^−1^). Using *V* = *M*/*ρ* and rearranging Equation (12) gives:

(13) $$\;\frac{\Delta Q_{\mathrm{ext}}}{V}=\frac{\rho \Delta H_{\mathrm{sub}}}{M}=\Delta P=\frac{\Delta F}{area}\approx \Upsilon_{\mathrm{solid}}-\Upsilon_{\mathrm{gas}}.$$

Because ϒ = 0 for gas, only Young’s modulus of the solid pertains, so the subscript is dropped. As in modeling expansion, force is presumed proportional to rigidity, yielding:

(14) $$\frac{\rho \Delta H_{\mathrm{sub}}}{M}=\mathrm{constant}\;\#2\;\times \Upsilon .$$

Proportionality constant #2 likely differs from constant #1 in Equation (11), since bonds are broken during sublimation, rather than being incrementally stretched as in thermal expansion.

The above analysis describes free sublimation in all three spatial directions. However, experiments often record products evolved from a surface, not from a volume. Thus:

- Constant#2 should be near ⅓.

### 2.3. Available Information on Sublimation from Previous Studies

Bonds are broken when a solid sublimates in order to release of atoms or ions from the surface. Surface and bulk properties need not be identical. Moreover, a thermal gradient exists at a surface including under presumed isothermal conditions because thermal conductivities of two materials composing an interface are rarely identical. Mass diffusion occurs in this thermochemical gradient. Sublimations depend on time up until steady-state conditions are reached, where back reactions occur at the surface. The terrace–ledge–kink model [19] is a popular description of this process [20].

The need for high temperatures to release measurable quantities of gas from most materials further complicates matters. The summary of measurements below is not comprehensive, but illustrates current knowledge. We discuss complexities that accompany quantifying the sublimation process, where ambiguities arise, and the high likelihood of irreversibility and disequilibrium conditions complicates matters, although steady-state is possible.

Despite the complexity, enthalpy of sublimation is represented by a single numerical value. This is in accord with classical thermodynamics being a macroscopic theory.

#### 2.3.1. How Sublimation Enthalpy Is Determined

As summarized by Shakeel et al. [21] and references cited therein, sublimation energetics are measured both directly and indirectly. Both approaches measure sublimation from a surface, not from a volume.

Direct techniques use calorimeters to measure heat uptaken (e.g., [22]). Indirect methods measure the vapor pressure at different temperatures and assume that Δ*H*_sub_ remains constant within the temperature range explored (e.g., [23]). Experimental uncertainties are generally considered to be below ~1%: See, e.g., the NIST database [17], which we utilize (Section 3). However, discrepancies between studies can be large, e.g., [24]. Moreover, results for free sublimation differ from effusive experiments that typically use a Knudsen cell [20,25]. Whether or not back transformation exists, and additional reactions occur in the gas phase, affect Δ*H*_sub_ extractions. The actual gas species released from the surface is crucially important, as this governs the reaction and its kinetics. Data analysis clearly requires making some assumptions, described as follows:

#### 2.3.2. Assumed Reactions and Reference States

Importantly, Δ*H*_sub_ values are model dependent, since a reference state is essential, along with specifying the reaction products. It is generally assumed at a phase transition that the change in Gibb’s free energy is 0, and hence that Δ*S* = Δ*H*/*T*. Entropy is not directly measured. Because calculations of entropy are removed from the measurements, the present report is limited to enthalpy.

**Figure 2:**
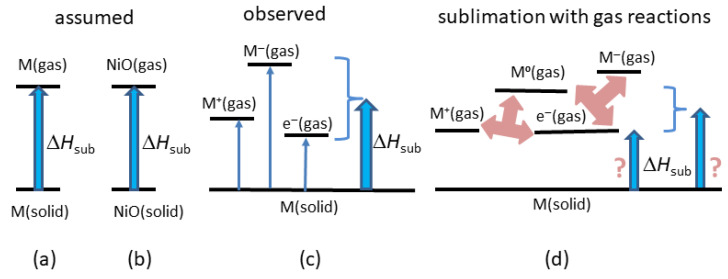
Schematics of sublimation reactions: (**a**) Assumed reaction describing metals and elements. (**b**) Assumed reaction describing compounds. (**c**) Mass spectrometry data on refractory metals (W, Ta, Re, Mo) [27]. (**d**) Two possible steady-state reactions in the evolved gas of univalent positive metal cations and electrons. The electrons may be in the gas, or on the surface. Multiplicity or variability in the reactions contributes ambiguity to enthalpy calculations, as illustrated.

The conventional approach in extracting Δ*H*_sub_ is to consider elements in their standard state. Alternatively, free atoms can serve as the reference state [26]. In any case, the specifics of the reactant and product(s) affect the calculation, as follows:

Structure and chemical stoichiometry of the solid are known. In most cases, sublimation of an element is assumed to yield neutral atoms whereas sublimation of a compound is assumed to yield neutral molecules (Figure 2a,b). This assumption, if incorrect, introduces systematic errors. Thus, Δ*H*_sub_ values may be precise rather than accurate. We return to these concerns in the discussion (Section 5).

#### 2.3.3. Mass Spectrometry and Other Determinations of Gas Constituents

Gas species evolved during sublimation of certain elements and compounds have been ascertained mostly via mass spectrometry. Such studies indirectly extract Δ*H*_sub_ from vapor pressure determinations. The examples below represent material types investigated in Section 4.

Mass spectrometry studies detect charged species. Hence, electron streams are applied which generally create positive ions and a negatively charged surface. These measurements probe the induced charge state. However, the charge state prior to applying the electron stream is equivocal. It is well known from isotope mass spectrometry that not only is the charge state of the gas constituents affected but the speciation (e.g., H, H_2_, H_3_ proportions) may be altered as well (R.E. Criss, personal communication). In studying gas molecules, increasing voltage of the electron stream produces a greater number of ions, more and different fragments [28] (p. 370). Even so, two key results point to ions being important, regardless of electron beam use:

- Modern mass spectrometry studies of molecular solids reveal that ions are produced spontaneously at room *T* and low *P* conditions in an evacuated chamber (e.g., [7,29]). Supplying additional energy or forcing production of ions (e.g., by lasers or electron bombardment) is not needed. High charge states (+7 to 14) have been observed for large molecules (e.g., Ubiquitin, a 76-amino acid protein) as well as negative ions [30].
- From a different (electrostatic) method in which electrons are not supplied, ice freely sublimates H^+^ in greater abundance than OH^−^ [31]. This proportioning is caused by surface *T* being colder than interior temperatures in these experiments. When the surface is warmer, negative charge is carried away, leaving a positive surface [31]. Neutral H_2_O and large, charged particles have also been observed in the gas [32].

Additionally and importantly, mass spectrometry shows that clusters of atoms are commonly evolved, except for metals. In the following examples, charge state is ambiguous, and is noted if specified by the authors:

- Positive and negative, singly charged ions have been observed to sublime freely from 4d and 5d transition metal surfaces, see the review of Scheer [27]. A greater number of positive than negative ions were observed for tungsten, rhenium, molybdenum, and tantalum. Only positive ions were detected for niobium.
- The metalloid of gray selenium subliming over 102 < *T* < 187 °C yields all possible Se_n_^+^ ions between *n* = 1 to 8 in measurable quantities [33]. Most common are Se_6_ and Se_2_. The solid structure includes spiraling chains. Similarly, insulating rhombic sulfur sublimates to S_8_, S_6_, and S_7_ [34].
- For semi-metallic graphite, sublimation provides C^+^ ions plus C_2_ and C_3_ positively charged molecules in roughly equal amounts [35]. Sheer [27] stated that C^−^ is detected. Similarly, clusters were observed for sublimating Si and Ge [36,37].
- Covalently bonded SiC, in its common hexagonal form at moderate *T*, predominantly sublimates to the gaseous species of Si, with slightly lower, subequal amounts of SiC_2_ and Si_2_C [38]. Dimers of Si are produced in even lower quantities. Solid graphite is a product. At high *T*, C, C_2_ and C_3_ are sublimated, as well as large molecules such as Si_2_C_3_. The products are sometimes referred to as ions, and sometimes as molecules by [38].
- Ionically bonded Li_2_O sublimates Li^+^, Li_2_O^+^, LiO^+^, and O_2_^+^ [39].
- Free surface sublimation of CsI yields Cs^+^ > Cs_2_I^+^ and small amounts of Cs_2_^+^ > CsI_2_^−^ > I^−^ at 877 K [20]. In contrast, gas effused during Knudsen cell experiments has balanced currents of positive and negative ions [20].

Clearly, clusters are produced, although the proportions of the species are uncertain to various degrees.

Lastly, Farber and Srivastava [40] examined data from weight loss studies of NiO, and concluded that sublimation evolves Ni + ½O_2_, which substantially exceed NiO gas. Species produced from sublimating other diatomic oxides are debated [41].

**Figure 3:**
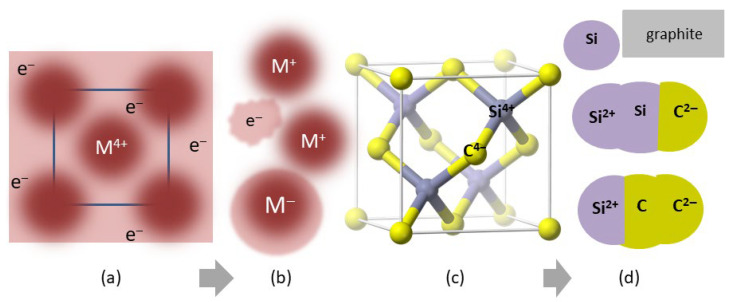
Schematics of changes during sublimation (gray arrow) observed in mass spectrometry: (**a**) Face-on view of a body centered cubic metal. Burgundy spheres represent cations that are surrounded by conduction electrons (the nearly free electron model). The metal cations studied (W, Mo, Ta) nominally have a high charge state. (**b**) Gas sublimated from W, Mo, Ta, and Re [27] where free electrons may be evolved but may be associated with the surface. (**c**) Projection view of the zincblende structure adopted by many chalcogenides, which describes cubic (3C) SiC. Coordination is tetrahedral. Yellow balls = C^4−^ anions. Purple balls = Si^4+^ cations. Fat sticks = bonds. (**d**) Gas sublimated from SiC at low *T* [38]. Charge states are ambiguous due to covalent bonding and use of electron beams in mass spectrometry. The most abundant gas species are shown, along with production of solid graphite [38]. Part (**c**) was modified after https://commons.wikimedia.org/wiki/Category:Zinc_sulfide, which is publicly available, and was accessed 26 May 2025.

Figure 3 provides a schematic of reactants and sublimation products of metals and of SiC at low *T*. Sublimation models for these and other materials studied using mass spectrometry are complicated. An electron gas suggested by charge conservation is not considered. More consistent with the above results is that the surface during free sublimation is negatively charged, since objects shed heat from their surfaces to the surroundings. Per Latham and Stow’s [31] ice experiments, an interior that is warmer than the surface yields positive ions, as observed for spontaneous sublimation of molecular solids [32], deduced for several metals [27] and free sublimation of CsI [20].

#### 2.3.4. Summary of Observations

As illustrated in Figure 2 and Figure 3, the observed products are more complex than the common assumption that the sublimated gas has a chemical formula identical to that of the parent solid. Additional ambiguity arises since components in the evolved gas may also react, especially at high temperature (Figure 2d). Are these reactions part of the heat uptake during sublimation? Does free sublimation inherently differ from Knudsen cell determinations?

After comparing sublimation enthalpy to elastic properties, we will return to questions regarding process. One goal is to better understand mechanisms.

## 3. Methods

### 3.1. Available Data Abounds

Previously published data are used. Elastic moduli and density at ambient conditions are largely from compilations, and are publicly available for many elements and compounds [16]. Generally, individual entries (e.g., [42]) were used. Data on radioactive elements are mostly insufficient for comparison. Primary sources were consulted as cross checks and to expand the database. Citations are found in the figures of Section 4. Uncertainties of ~1 to 2% in ϒ are smaller than symbol size in Figure 1 and those subsequently shown, unless noted otherwise.

Sublimation enthalpies were mostly obtained from the difference between the enthalpy of formation for the solid and the enthalpy of vaporization for its gas, using the National Institute of Standards database on thermochemical properties [17]. This database is largely based on measurements compiled by Chase et al. [43]. As discussed in Section 2.3.1, experimental uncertainties in Δ*H*_sub_ are generally considered to be below ~1%, but discrepancies exist. Error bars for this compilation are smaller than symbols in the figures below. Decomposition to neutral species generally is assumed. Molecular weights are also listed, whereas density is listed in sources for elastic data. For molecular solids and a few other compounds, additional sources for sublimation enthalpies are noted in Section 4.

### 3.2. Comparisons of Energy Density to Elastic Moduli

Sublimation enthalpy (Δ*H*_sub_) is typically reported in J mol^−1^, which converts to J g^−1^ by dividing Δ*H*_sub_ by molecular mass, or to J m^−3^ for the product *ρ*Δ*H*_sub_/*M*. All three representations are valid and depict energy associated with the average neutral species from different perspectives. We focus on the product *ρ*Δ*H*_sub_/*M* because this describes occupation of space by energy whereas *Y* describes how the solid is rigidly held in that very same space. We also compare Δ*H*_sub_/*M* to *ρ*ϒ (J g^−1^) and Δ*H*_sub_ to *ρ*ϒ/*M* (J mol^−1^) to probe mass and density effects.

Connections of sublimation enthalpy to bulk modulus, shear modulus, and Poisson’s ratio are mostly explored on a per volume basis. All comparisons trace to per mole of the neutral species, i.e., to the element or to the formula for the compound.

## 4. Results

### 4.1. Metallic Elements Grouped According to Crystallographic Structure

Similar chemical bonding in metallic elements permits isolating the effect of structure. Many metals have either the face centered cubic (fcc), or body centered cubic (bcc), or hexagonal close packed (hcp) structure. Although hcp is not cubic, each atom has 12 nearest neighbors, as in fcc.

#### 4.1.1. Three Comparisons of Sublimation Enthalpy to Young’s Elastic Modulus

**Figure 4:**
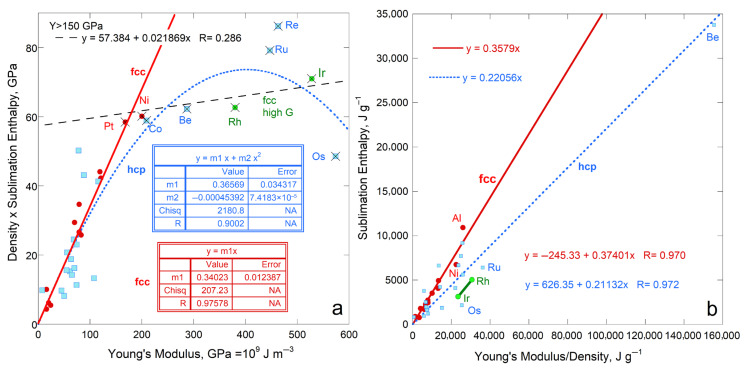
Comparison of Δ*H*_sub_ on a per-atom basis to ϒ, which involves *ρ*, for fcc and hcp metallic elements. Both structures have 12-coordinated atoms: (**a**) Energy density per volume; (**b**) Energy density per mass. For both panels, least squares fits are given, where NA signifies Not Applicable. Red dots = fcc, except for the two metals with the highest shear moduli (green dots). These two metals are included with the high-ϒ hcp samples (×) in fitting in part a (dashed line). Blue open squares are hcp metals, where the fit to all samples in part b is largely controlled by one metal, Be. The fcc samples Rh and Ir are like the high-ϒ hcp metal, but are not include in fitting. Data are publicly available [16,17], where reported uncertainties are smaller than symbol size. See Section 2.3 and Section 3 for further discussion. All fcc and hcp elements with data on ϒ are included.

Sublimation enthalpy on a per volume basis for the 14 fcc elements depends linearly on ϒ up to 200 GPa (Figure 4a). The four most rigid fcc metals define a second linear trend with a lower slope. Available data on 21 hcp elements are scattered about the fcc trends at each of low and high ϒ. Elements Rh, Ir, Ru, Re, and Os with high ϒ have high *G* (exceeding 150 GPa) and high melting points (above 2239 K). Possibly, at high *T* needed to sublimate refractory elements enhances ductility which offsets resistance to shear.

Considering energy density per mass likewise provides a linear trend for fcc metals, again excepting Rh and Ir with high ϒ and *G* (Figure 4b). The slope and scatter are similar to per volume (Figure 4a) but low density (light) elements greatly influence the per-mass fits (Figure 4b) The hcp elements roughly follow the per-mass trend for the fcc elements with low *Y* and *G*, while the stiffer hcp metals fall near the trend for stiff fcc elements Rh and Ir. For hcp, beryllium controls the mass trend due to its very low density, which trend is otherwise scattered.

**Figure 5:**
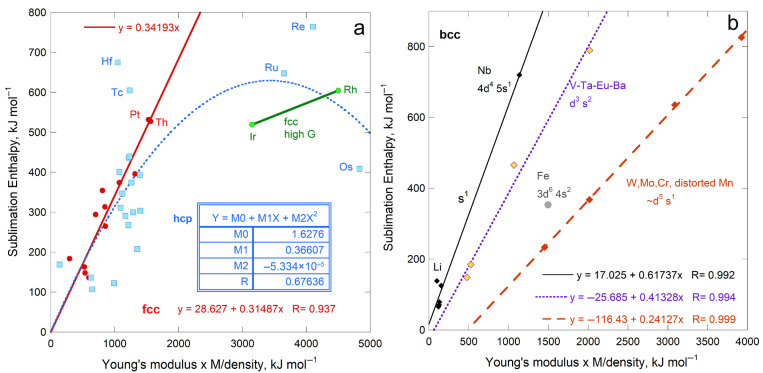
Comparison of ΔH_sub_ (as reported in kJ mol^−1^) to *M*ϒ/*ρ*. Least squares fits are shown: (**a**) Metals with 12-coordination. Red dots = fcc metals, except for the two metals with extremely high shear moduli (green dots), which were too few to fit. Blue squares = hcp metals; (**b**) Metals with 8-coordination (bcc structure). Black diamonds and solid lines = metals with a single s electron. Purple diamonds with yellow interiors are bcc metals with two s electrons and three d electrons. Orange diamonds are column VIB metals with similar electron configurations and Mn, which has a distorted structure. Data are publicly available [16,17], where reported uncertainties are smaller than symbol size. See Section 2.3 and Section 3 for further discussion.

Using measured Δ*H*_sub_ for the y-axis (energy per mole: Figure 5a) mimics the results for energy per volume for fcc and hcp metals. The proportionality constants are similar although the scatter is greater, particularly for hcp, as evident visually and in the residuals of fitting, *R*.

**Figure 6:**
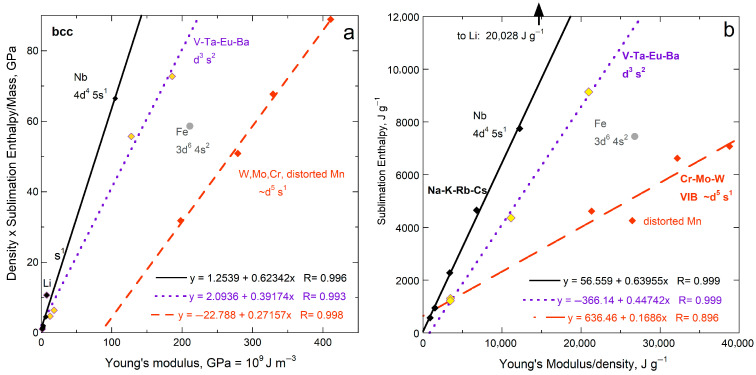
Comparison of Δ*H*_sub_ on a per-atom basis to ϒ, which involves *ρ*, for bcc metallic elements, which structures has 8-coordinated atoms. Least squares fits are shown; (**a**) Energy density per volume; (**b**) Energy density per mass. Least squares fits are shown, with and without an intercept. Filled diamonds and solid lines = the alkali metals except for Li. Purple open diamond are Li and other hcp metals, as labeled. Data are publicly available [16,17], where reported uncertainties are smaller than symbol size. See Section 2.3 and Section 3 for further discussion. The few (radioactive) bcc elements lacking data on ϒ and/or *G* could not be included.

For all 15 bcc elements, plots for all three representations of energy density seem scattered, yet systematic behavior exists (Figure 5b and Figure 6a,b). The alkali metals (IA) with their single s electron, excluding lithium, form steep trends with negligible scatter. Niobium, with its single s electron, falls near the IA trend. The five bcc elements with ϒ > 186 GPa (Ta, Mn, Fe, Cr, Mo, W) define trends in accord with similar electronic configuration (Figure 5b and Figure 6a,b). The groupings are likely connected with greater variation in force constants for bcc, because atoms in the bcc structure have eight nearest neighbors at 0.866 *L*, while six 2nd-nearest neighbors existing at *L* are quite close. Secondary bonding is thus more substantial in bcc metals than in the fcc and hcp with 12 nearest neighbors. Secondary bonds are likely affected by differences in electronic configurations.

#### 4.1.2. Poisson’s Dimensionless Ratio

**Figure 7:**
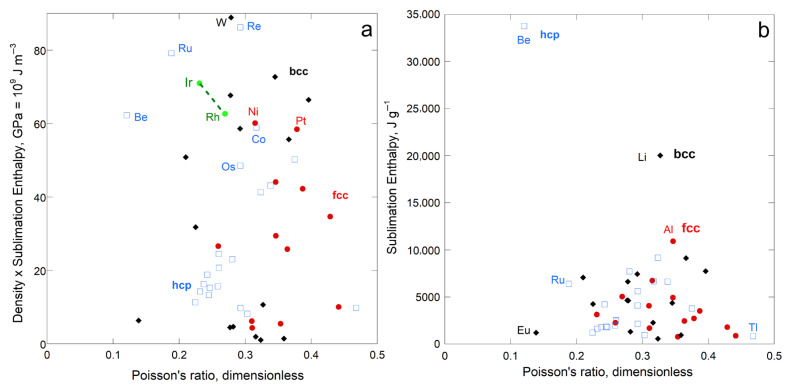
Comparison of Δ*H*_sub_ to Poisson’s ratio for metallic elements. A correlation does not appear to exist for any sub-type, so fits are not provided. Red dots = fcc, except for the two metals with the highest shear moduli (green dots). Blue open squares are hcp metals. Black diamonds = bcc: (**a**) Presentation as energy per volume; (**b**) Presentation as energy per mass. Data are publicly available [16,17], where reported uncertainties are smaller than symbol size. See Section 2.3 and Section 3 for further discussion.

No correlation was found between sublimation enthalpy per volume and Poisson’s ratio for the fcc, hcp, or bcc element groups, either individually or together (Figure 7a). Hence, distortion is unimportant during sublimation. Figure 7b shows that light atoms strongly influence the comparison of enthalpy with ν when cast as energy per mass, as surmised for rigidity (Figure 5b and Figure 6b). The per-mole representation for ν is scattered like the per volume representation of Figure 7a, and so is not shown.

#### 4.1.3. Comparisons of Sublimation Enthalpy to Shear Moduli

**Figure 8:**
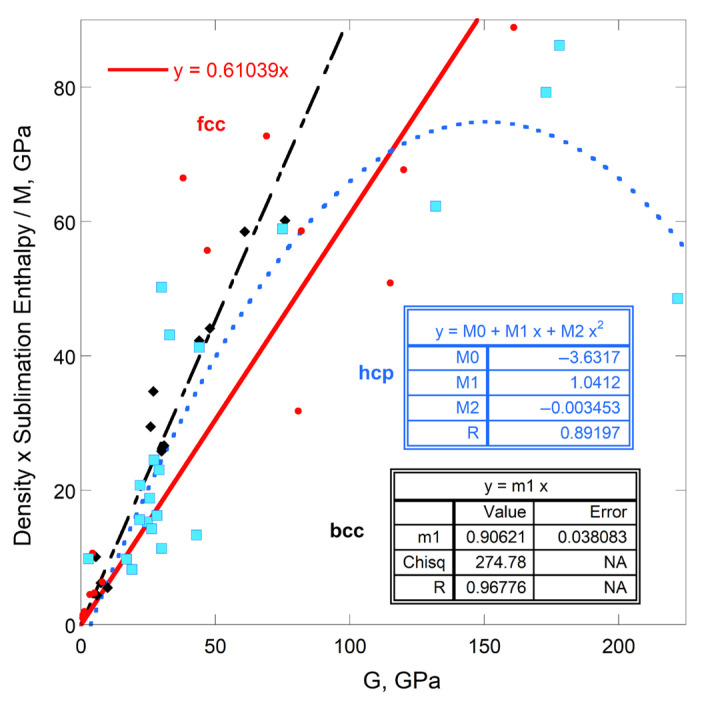
Comparison of sublimation energy per volume to shear modulus of metallic elements. Red dots = fcc; blue squares = hcp; black diamonds = bcc metals, including Li. Least squares fits are shown, where NA indicates Not Applicable. Data are publicly available [16,17], where reported uncertainties are smaller than symbol size. Section 2.3 and Section 3 provide further discussion.

Shear moduli of fcc, bcc, and hcp metals correlate with Young’s modulus (Figure 1a). Thus, *ρ*Δ*H*_sub_/*M* depending on ϒ means that enthalpy should also depend on *G*. Instead, a linear correlation with a reasonable residual is only observed for the bcc structure (Figure 8). Significantly more scatter exists about the trends of *ρ*Δ*H*_sub_/*M* with *G* for fcc and hcp metals (Figure 8) than with *Y* (Figure 4a). This is particularly true for fcc metals.

The connection of sublimation with *Y* for fcc and hcp structures, but with *G* for bcc metals is ascribed to the importance of the 2nd nearest neighbors to forces in bcc, which array provides forces in directions other than radial about a given atom, thereby resisting shear. Interestingly, the electronic configuration is unimportant to the connection of enthalpy with *G* for bcc metals (Figure 8) but had a strong effect on the dependence of sublimation enthalpy on ϒ (Figure 6a). The different trends in Figure 6a thus arise from variations in the shear modulus, which depends on electronic configuration and other factors.

Plots of energy density on a per-mass or per-mole basis for *G* are similar to Figure 8. These plots are not shown because scant additional information is provided.

#### 4.1.4. Comparisons of Sublimation Enthalpy to Bulk Moduli

**Figure 9:**
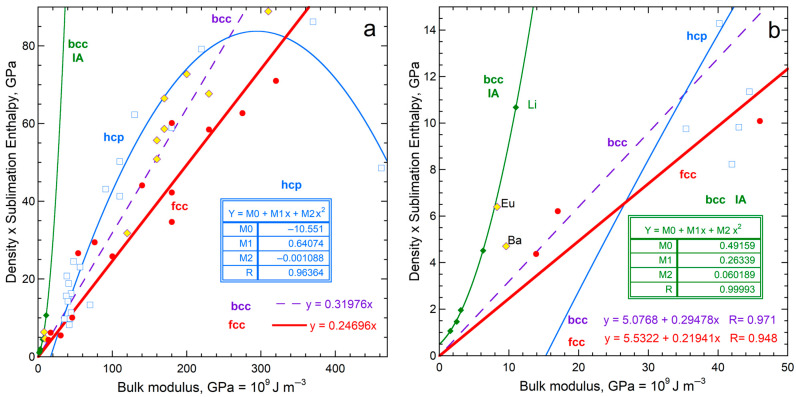
Comparison of *ρ*Δ*H*_sub_/*M* (energy per volume) to bulk modulus of metallic elements. Various least squares fits are shown: (**a**) Full view; (**b**) Expanded view near the origin. Red dots = fcc; blue squares = hcp; green diamonds = bcc alkali metals, including Li; purple diamonds with yellow interiors = the remaining bcc elements. Data are publicly available [16,17].

Sublimation enthalpy on a per volume basis correlates roughly with bulk modulus (Figure 9). For fcc metals, the fit with *B* is poorer than the fit with ϒ (Figure 4a). Fits for the bcc alkali metals (IA) include lithium, but the correlation is non-linear and the origin is not included (Figure 9b). For bcc elements other than alkali metals, sublimation energy correlates with bulk modulus reasonably well, albeit with scatter. For hcp metals, less scatter exists in the fit to *B* than with the fit to ϒ (Figure 4a), but the intercept is far from the origin and negative (Figure 9a,b).

Plots of energy density on a per-mass or per-mole basis involving *B* are similar to Figure 9. These plots are not shown because little additional information is provided.

### 4.2. Summary of Metal Behavior and Sufficiency of the Energy Density Representation

The above comparisons for fcc metals show that sublimation enthalpy on a per volume basis correlates directly with Young’s modulus. Figure 9, in conjunction with Figure 4, Figure 5, Figure 6, Figure 7 and Figure 8, confirms that the bulk modulus is most important to sublimation but rigidity and resistance to shear play essential roles. Although the hcp structure is anisotropic, trends are similar to those for fcc, which likewise has 12-coordination. The correlation with ϒ holds roughly for the bcc metals, for which structure interatomic forces depart further from spherical symmetry (8 vs. 12 coordination), and for which shear forces, 2nd nearest neighbors, and electronic configurations play important roles.

Portrayal of sublimation enthalpy on a per volume basis provides the best fits and salient information for all the elastic moduli. Considering a per-mass basis points to density having some effect, particularly when low. Using a per-mole basis provided trends differing little from the per volume basis.

Given the results in Section 4.1, summarized above, the comparisons below consider only the dependence of *ρ*Δ*H*_sub_/*M* on ϒ. This approach permits focusing on effects of chemical bonding, more complicated structures, and anisotropy.

### 4.3. Elements Occupying Vertical Columns in the Periodic Table

Groups in the Periodic Table have similar chemical bonding but few members. Simultaneous restrictions on structure and bonding provide the well-defined trend of *ρ*Δ*H*_sub_/*M* with ϒ for the bcc IA alkali metals (Figure 6a).

**Figure 10:**
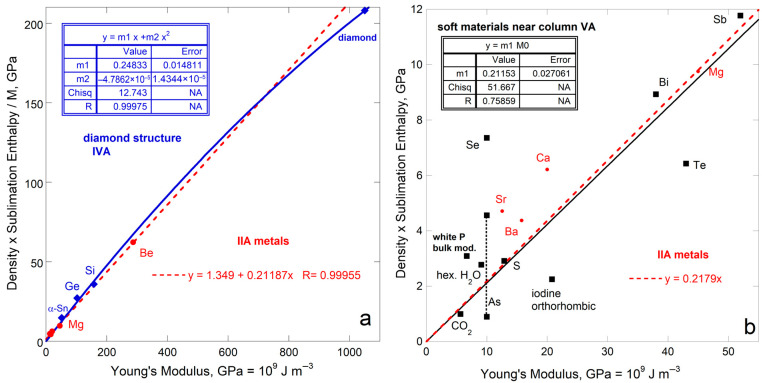
Comparison of *ρ*Δ*H*_sub_/*M* (energy per volume) to Young’s modulus for various elements and ices. Least squares fits are shown, where NA signifies Not Applicable. Blue diamonds = diamond structure. Red dots = alkali earth metals. Black squares = soft elements and ice compounds: (**a**) The IVA diamond structure for which bonding varies and the IIA alkali earth metals for which structure varies. (**b**) Soft V elements, along with ices, fit to one trend (black symbols and line). Also shown are the softest of the IIA metals (red dots where high-*Y* data are also fit). Publicly available data [16,17] were supplemented with lower bound on arsenic sublimation from [25], and additional data on elements from [44,45,46,47]. Ice data from [21,48,49].

The only other isostructural group with several members is the four elements in the IVA column with the diamond structure. This group includes one insulator (C), two semiconductors (Si, Ge) and one metal (α-Sn). Diamond’s immense ϒ controls the fit of *ρ*Δ*H*_sub_/*M* to ϒ*,* which is best described by a polynomial with an intercept of 0 (Figure 10a). Excluding diamond provides a roughly linear dependence of *ρ*Δ*H*_sub_/*M* on ϒ. However, the fit to Si, Ge, and α-Sn is equivocal because both *G* and ϒ are uncertain for Si. Behavior of group IVA mimics that of metallic elements, but departures from a linear correspondence with *ρ*Δ*H*_sub_/*M* occur at much higher ϒ for these brittle materials than for ductile metals.

The 5 alkali earth metals of Group IIA have various structures. The observed trend (Figure 10a) is controlled by light hcp Be and hcp Mg, while bcc Ba and fcc Sr and Ca diverge. See the expanded view of Figure 10b.

The members of Column VA (P, As, Sb, Bi) include metals and non-metals. Bismuth has a rhombohedral layered structure like the metalloids antimony and arsenic. Sublimation enthalpy links to Young’s modulus, with divergence increasing as *ρ*Δ*H*_sub_/*M* decreases (Figure 10b). The fit is weak because *G* is lacking for phosphorous while *ρ*Δ*H*_sub_/*M* is highly uncertain for arsenic. Nonetheless, non-metals in the adjacent VIA column (S and I_2_) fall near this trend as do the well-studied ices (H_2_O and CO_2_). Ices are included in Figure 10b because O is a column VIA element and these solids are covalently bonded like As, P, S, and I_2_. The soft metals Se and Te in IVA fall far from the trend. Structures of these elements include chains or rings or helixes. Mass spectrometry measurements (Section 3.2) and thermodynamic studies [25] indicate the gasses include variously sized molecules. Thus, consistent reactions do not exist for the elements explored in Figure 10b. Water ice likewise sublimates multiple species, but in a much more restricted fashion [31] and differs marginally from the soft element trend. The strongly covalent bond in CO_2_ suggest sublimation releases this molecule, which simplicity explains this ice lying on fit, largely defined by the metals Sb and Bi.

With both structure and bonding varying, plus uncertainties existing in the measurements, the link between sublimation enthalpy and rigidity is weaker, but still exists. Figure 10 shows that structure has a greater effect on the link of *ρ*Δ*H*_sub_/*M* to ϒ than does chemical bond type.

### 4.4. Simple Compounds with Mostly Cubic Structures

For compounds, sublimation enthalpy on a per-mole basis refers to the formula unit, rather than to the atom, as in the elements. Many diatomic compounds crystallize in simple structures.

#### 4.4.1. Isostructural, Ionically Bonded Oxides and Halides

Simple oxides (XO, where X is a metal cation) crystallize in the B1 structure. This (rocksalt) structure can be described as an fcc lattice of cations with anions occupying each octahedral void or vice versa. One type of chemical bond exists between the 6-coordinated atoms.

**Figure 11:**
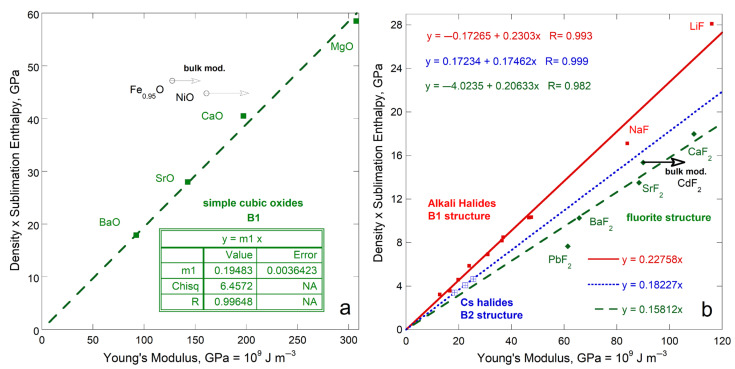
Comparison of *ρ*Δ*H*_sub_/*M* (energy per volume) to Young’s modulus for simple compounds with cubic structures. Least squares fits are provided where NA indicates not applicable: (**a**) Oxides in the B1 structure; (**b**) Halides in B1, B2, and fluorite structures. Most of the elastic constants were calculated from the compilations of *B* and *G* by [14] and/or by [50,51]. Enthalpies from [17,52].

For B1 oxides, sublimation enthalpy (per volume basis) linearly depends on ϒ (Figure 11). Sublimation of NiO evolves mainly Ni and ½O_2_ gas [40]. Location of NiO above the trend defined by the other oxides suggests that sublimation products of column IIA metal oxides differ from those of NiO: Molecules are mostly produced, as is generally assumed. Wüstite, like NiO, lies above the trend for IIA metal oxides and likewise contains a transition metal. We suggest that Fe_x_O likely sublimates mostly Fe metal plus ½O_2_ gas.

Linear correlations also exist for the well-studied alkali halides in the B1, B2, and fluorite structures (Figure 11b). The B2 (CsCl) structure has 8-coordination as in the bcc elements. The fluorite structure has 4-coordinated cations and 8-coordinated anions. Only one type of chemical bond exists in all three cubic structures, which is strongly ionic. As occurred for the simple oxides, compounds with multi-valent cations (Pb) and transition metals (Cd) depart from the trends of the cations with a single charge state (loss of two s electrons). Trends of sublimation energy density with *B* or *G* differ little from the relationship with ϒ, shown in Figure 11b.

#### 4.4.2. Diatomic Semiconductors

**Figure 12:**
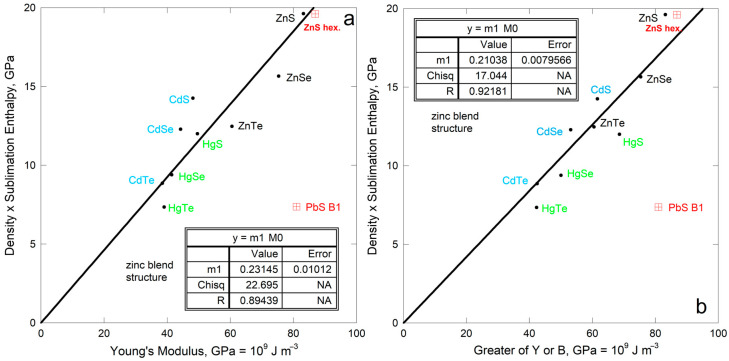
Comparison of *ρ*Δ*H*_sub_/*M* (energy per volume) to elastic moduli for chalcogenides. Data from [26,53] (pp. 41–72). Colored labels are grouped according to cations. Red symbols show compounds excluded from the fit for structural differences. Least squares fits are provided where NA indicates not applicable: (**a**) Dependence on ϒ. (**b**) Dependence on the greater of ϒ or B.

The IIB-VI compounds crystallize in various structures. Enthalpies of sublimation were compiled by [26], five of which compounds possess the cubic zinc blende structure (Figure 12a). Elastic properties were compiled by [53].

The trend describing the isostructural chalcogenides also applies to the entire group. The bonding is mostly ionic, but not entirely. Thus, the scatter is attributed to variations in gas species evolved which stem from bonding differing among the isostructural compounds. Note that similar slopes are seen for all compounds examined which have one type of bond (Figure 11a,b and Figure 12).

Comparing sublimation energy density to the greater of Young’s or bulk modulus improves the fit. Also, compounds with the same cations occupy distinct trends. Variations in the degree of ionic vs. covalent bonding are implicated.

### 4.5. Molecular Solids

Elastic data on about 20 molecular solids have been studied multiple times: yet disagreements exist. We rely on the review and assessment by Spackman et al. [54]. Likewise, although data on sublimation of molecular solids are ample, disagreements are significant. We rely on multiply studied substances as reviewed by Chicos et al. [55].

**Figure 13:**
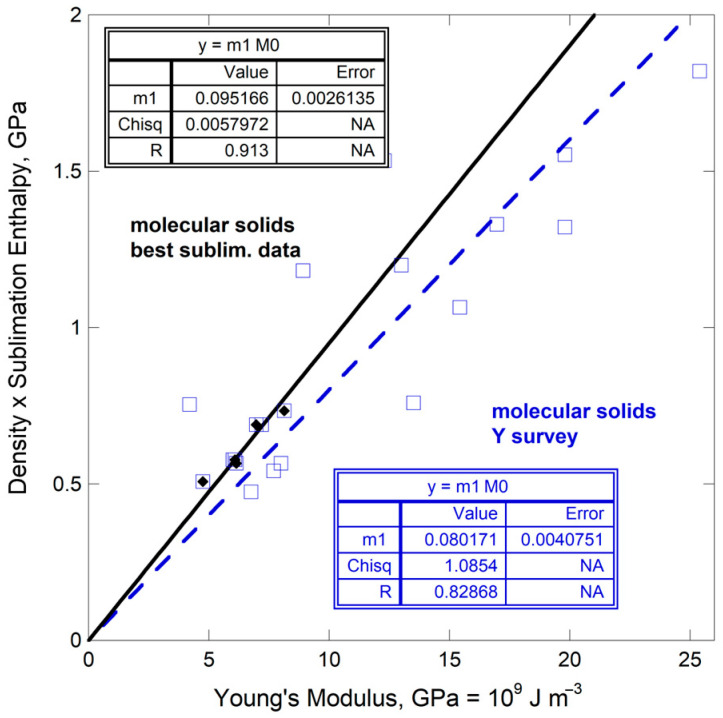
Comparison of *ρ*Δ*H*_sub_/*M* (energy per volume) to Young’s modulus for molecular solids. *Y* data from [54]. Black diamonds = enthalpy data from [55]. Blue squares = enthalpy data from [56]. Least squares fits are provided where NA indicates not applicable.

Only five compounds are common to both reviews: anthracene, benzene, benzophenone, naphthalene, and 2-hydroxybenzoic acid. The dependence of *ρ*Δ*H*_sub_/*M* on ϒ is well-defined, albeit over a narrow range (Figure 13). This trend is similar to that defined by molecular solids with robust *Y* values [54] while using available data on *ρ*Δ*H*_sub_/*M* [56].

### 4.6. Proportionality Constants

Table 1 summarizes results of fitting sublimation enthalpy, cast as energy per volume, directly to ϒ. Table 1 also compares this proportionality constant to that describing the heat involved in expanding the solid, from [1]. For convenience, Equations (11) and (14) are repeated in the bottom row.

The two proportionality constants differ; yet, each occupies a limited range and both depend on the structure of the solid. Constant #2 for sublimation varies from 0.1 to 0.7. Constant #1 for expansion varies much less (0.33 to 0.6), although fewer material types were investigated. The following trends are evident from Table 1:

- On average, the energy per volume required to sublimate fcc, hcp, and bcc metals is about ⅓ of Young’s modulus. For metals with the bcc structure, constant #2 varies widely, depending on the electronic configuration;
- On average, the energy per volume required to sublimate various other types of solids is ~0.2 times Young’s modulus. These types consist of (1) elements with the diamond structure (few bonds, so spherical symmetry is violated), (2) elements in columns VA and VIA plus ices, and (3) many different diatomic compounds with one type of chemical bond (generally ionic);
- Regarding the compounds, sublimation does not break all bonds (Figure 2). The primary bond remains. Hence, less energy is needed to overcome the internal resistance when converting a solid compound to a gas of molecules, compared to converting an element to a gas of atoms. Consequently, the proportionality constant is reduced from ~⅓ to ~⅕;
- Sublimating the molecular solids only requires breaking weak molecular or hydrogen bonds. Hence, much less energy is needed.

## 5. Discussion

### 5.1. Sublimation Enthalpy Depends Unequivocally on the Rigidity of Solids

Theoretically, the heat-energy needed for sublimation depends linearly on Young’s modulus (Section 2.2.2). Pressure units are equivalent to energy per volume, but ϒ actually represents the longitudinal stress–strain response of an elastic solid (Equation (4)). We have argued that *ρ*Δ*H*_sub_/*M* = ⅓ϒ applies, since sublimation is directional. Namely, gas is released from a surface, not from a volume.

Our theory is validated for diverse types of solids by the proportionality constant being on the order of ⅓ (Table 1). Verification utilized >100 elements and compounds and mostly, but not entirely, was based on cubic structures. This choice avoids complications of the orientation rarely being stated in sublimation experiments, while still providing a large database with diverse structures and chemical bond types.

Importantly, *ρ*Δ*H*_sub_/*M* is significantly less than ϒ for all cases examined. This relation shows that the vibrational energy stored in the solid, which is related to the temperature of the body, has negligible effect on sublimation. Young’s modulus and density depend weakly on *T* for most solids, including those studied here (see figures in [1]). Hence, the trends shown in Section 4 are little impacted by *T* used in determining Δ*H*_sub_.

Departures from a linear relationship and variations in the proportionality constant are related to structure and bonding, since the best fits were found for series with similar chemical bonding or electronic configuration. Details follow in Section 5.2.

#### 5.1.1. Verification Depends on Accuracy

Measurements of Young’s modulus are accurate. Not only can ϒ be directly measured, but for cubic structures, ϒ is furthermore simply related to bulk and shear moduli (Equation (5)). This cross-check is robust because *B* and *G* have been multiply measured in various ways of a very large number of solids (see, e.g., [1,15,50]). For the few non-cubic structures considered here, averages are constrained, by well-known relations and substantial databases (e.g., [15,16,54]).

In contrast, measurements of Δ*H*_sub_ are uncertain, much more than generally recognized, due to several factors covered in Section 2.3. Key issues are:

- The evolved gas molecules consist of several different types of clusters for the various non-metallic elements and compounds, as well as for very soft metallic elements (Se and likely Te). Speciation is not always known and so is not always accounted for. Speciation is uncertain because neutral gas constituents are not detected in mass spectrometry. Yet, speciation affects the amount of heat needed to produce the gas (see below).
- Free sublimation gives different enthalpies than effusion from a Knudsen cell [20], which describes most measurements. Disagreements also exist between studies for unclear reasons.
- Measurements are made mostly at high temperatures where secondary reactions in the gas phase can occur, and might affect the amount of heat uptaken during sublimation.

The uncertainties and ambiguities in Δ*H*_sub_ contribute scatter to the trends in Figure 4, Figure 5, Figure 6, Figure 10, Figure 11, Figure 12 and Figure 13. Systematic relationships between the parent solid and gas produced are attributed to the proportionality constants for subsets of materials varying from ⅓ and the trends departing from linearity, as discussed in Section 5.2.

#### 5.1.2. Relationships with Other Elastic Moduli

Poisson’s elastic modulus does not directly pertain to sublimation (Figure 7). Sublimation enthalpy depends directly on *G* only for the bcc structure, despite the strong correlation of shear moduli with ϒ (Figure 1). This link stems from the nature of bcc atomic arrangements (Section 5.2.2). Bulk moduli less strongly depend on ϒ (Figure 1) and are less strongly related to sublimation.

Thus, the large volume change does not control sublimation. Instead, rigidity of the solid is most important to the energetics of sublimation.

### 5.2. Systematic Variations in the Dependence of Sublimation Enthalpy on Young’s Modulus

#### 5.2.1. Effects of Ductile vs. Brittle Behavior at High Temperature

Departures from linearity (e.g., either a polynomial fit or scatter) occur at high G. When stiff materials exist in a class are explored, the steep linear trend at low to moderate ϒ flattens at high ϒ, either providing a polynomial or a second, flat trend that is controlled by these stiff materials. The transition point of ~100 to 200 GPa and degree of flattening in ductile metals is far larger than those observed for insulators (oxides and diamonds) and by the brittle metal Be (Figure 4a, Figure 6a, Figure 10a and Figure 11a).

Because high ϒ is accompanied always by high *G*, the strong changes in metals are attributed to ductile behavior offsetting elastic resistance to shear. Compensating behavior occurs because materials with high ϒ are also refractory (e.g., [57]), so sublimation experiments are performed at high *T*, which enhances ductility. For the brittle IVA elements, the trend is a polynomial that is depends on diamond properties at high *T* and uncertain elastic data for Si.

Ductility and brittle behavior are also unique properties of solids. Their influence on trends of *ρ*Δ*H*_sub_/*M* with ϒ underscores that the process of sublimation is controlled by the parent solid, not by the gas produced.

The remainder of this section thus focusses on differences in the trends at low to moderate ϒ, where the elastic response of the metals governs their sublimation behavior. For many of the compounds explored, Δ*H*_sub_ data are at low to moderate *T*, which minimizes potential effect of gas reactions on heat uptake.

#### 5.2.2. Effects of Structure When the Chemical Bond Is Metallic

The best agreement of *ρ*ΔHsub/M with ⅓ϒ (excluding extreme values of ϒ for reasons covered above) occurs for the fcc and hcp metals. Mass spectrometry data shows metals evolve single atoms. For this case, all bonds are broken to release the gas. This behavior requires the energy stored in the solid be released as the surface is dispersed.

The bcc metals on average provide *ρ*Δ*H*_sub_/*M* ~ ⅓ϒ, but have a range of slopes, whereas the dependence of *ρ*Δ*H*_sub_/*M* on *G* is linear. Shear is more important to sublimation than in the fcc or hcp metals, as follows. In bcc structures, the 2nd nearest neighbors restrict shearing and thus contribute to rigidity. These are large in number (six) compared to the nearest neighbors (eight) and are relatively close. The importance of this secondary bonding depends on the electronic configuration. The alkali metals (IA) contribute their single s electron to the conduction band (Figure 3a) so these cations are closer to spherically symmetry than those with various charge states. Constant #2 for IA being very close to ⅔ (Figure 6a, Table 1) is attributed to the number of bonds being broken being about 2× that implicated by 8-fold coordination in a unit cell. Coordination in fcc and hcp is 12-fold and close-packed so these structures reasonably approximate spherically symmetric forces assumed in our derivation (Section 2.2.2).

#### 5.2.3. Behavior of Mostly Non-Metallic Group VA Elements and Group IIA Metals

For these classes of materials, *ρ*Δ*H*_sub_/*M* ~ 0.2ϒ (Figure 10). For the few substances studied by mass spectrometry, sublimation evolves clusters. For this case, less heat energy than that stored in the parent solid is needed to release gas, since not all bonds are broken.

Speciation of gas from IIA metals has not been determined. Both Be and Mg are brittle and control the slope for the group. The slope for ductile Ca, Sr, and Ba is ⅓, like that of other metals with low ϒ, which supports the above findings.

We propose that Be and Mg evolve clusters of atoms, due to their high strength. For Mg, rigidity is not connected with being refractory, but more with the small size of the Mg ion. Substances with small, light atoms (low density) uniformly diverge from the trends with ϒ defined by the larger, heavier atoms while controlling the trends of energy on a per-mass basis (Figure 4b and Figure 6b).

#### 5.2.4. Uniform Behavior of Compounds

For all simple compounds, *ρ*Δ*H*_sub_/*M* is near 0.2ϒ (Figure 11 and Figure 12; Table 1). This holds for all structures considered and whether bonding is ionic or covalent or mixed. Gases sublimated from simple compounds include multiple species that vary in complexity (Section 2.3). Clearly, not all bonds are broken, so less than the entire elastic energy of the solid must be overcome. For this reason, the slope is significantly below the inferred factor of ⅓. We suggest that the consistency is related to simplicity of the chemical formulae. Hence, a rather limited number of species are evolved to form the gas.

The effect of structure is evident in variations of constant #2 in Equation (14) from 0.2 (Table 1). Variations are most evident in comparing the ionically bonded halides and fluorites (Figure 11).

The heat of sublimation for molecular solids depends more weakly on ϒ than it does for the simple compounds. For molecular solids, the weak van der Waals bonding between the molecules is disrupted. Disruption should depend on orientation, which is not reported in sublimation studies, yet has an extreme effect on elastic properties [54]. Disruption should also depend on the size of the fragments in the gas, which was not investigated here, due to uncertainties in both Δ*H*_sub_ and ϒ for these non-cubic materials. We suggest that disparities between different experiments result from variations in fragments produced. Further investigation is warranted.

### 5.3. The Process of Sublimation

Our comparison of the enthalpy of sublimation to Young’s elastic modulus has shown that sublimation occurs via heat energy overcoming the elastic reservoir energy of a solid on the surface presented. For high-ϒ metals, an important factor is their ductility, which property offsets the elastic response to deformation at the high temperatures explored during sublimation of this refractory class. Otherwise, the correlation is strongly affected by the number, arrangement, and type of bonds which are broken, as described above. This connection provides insight into the process of sublimation.

#### 5.3.1. Gas Speciation Is Controlled by the Chemical Bond

When bonding is metallic, the solid consists of an array of cations surrounded by a nearly free electron gas (Figure 3a). Metals must have a negatively charged surface given evidence supporting the nearly free electron model [11] and that atoms are negative on their outsides. Clusters were not detected by mass spectrometry (Section 2.3). Hence, single metal atoms are released. The charge state is most likely neutral, which type of atom diffuses most readily in self-diffusion experiments [58]. But because the metals studied (Nb, Mo, Ta, W, Rh) typically have high charge states, any reduction in the positive charge would assist escape from the solid. We suggest that near-surface cations located at irregularities (defects, terraces, ledges), as previously recognized [19,20], combine with surface electrons to provide a neutral or singly charged atom that can escape. Some kinetic energy is lost in the process, so the evolved gas has a lower temperature during free sublimation. Adding heat to enhance yield alters this finding somewhat.

Insulators likewise should have negative charges at the surface, due to existence of dangling bonds (Figure 3c). Neutral species are most likely released, as in metals. Ionically bonded diatomic NiO apparently releases Ni and O atoms, more than NiO molecules. More heat-energy is needed to sublimate NiO or FeO, than the IIa metal oxides (Figure 12a), i.e., more bonds are broken, suggesting the IIa metal oxides form molecules mainly. This difference is attributed to bonding since Ni and Fe cations have d-electrons in the highest energy states (outermost), whereas IIA cations have closed shell configurations.

For covalent insulators, the bond is highly directional (Figure 3c), which promotes cluster production. Consequently, sublimation does not require the entire reservoir of elastic energy. The consistency of proportionality constants (Table 1) suggests that simple, neutral molecules are the dominant species.

#### 5.3.2. Isothermal Conditions?

All atoms in matter interact via collisions, which are long range in gases, but short range in solids. Because the electron clouds are soft and deform, these collisions have an inelastic component where some of the translational energy is lost in each collision [6] (pp. 143–179). This ubiquitous behavior of matter requires a heat source to maintain any body at some finite temperature.

Furthermore, thermal gradients always exist as described by Fourier’s theory. During sublimation, the atoms or molecules in the thermal boundary layer are lost. These are colder than the interior atoms (unless extra heat is supplied to the surface), and so the sublimated gas is colder than the solid and cannot uptake thermal energy from the solid per thermodynamic law. Only fast (hotter) gas constituents can return to the solid state because their kinetic energy must overcome the temperature difference and must suffice to bond (match the elastic energy).

#### 5.3.3. Equilibrium?

In discussing sublimation, McEwen et al. [7] pointed out that a sufficiently charged surface could produce a repulsive force that reduces the thermal energy necessary for ion emission, as in field desorption mass spectrometry [59]. Per Latham and Stow [31], positive ions are emitted for a surface cooler than the surroundings. Such a temperature gradient is required in evacuated environment, used to ensure purity of the evolved gas.

Surface gradients of charge and temperature need not be strong. But the charge gradient should inhibit return of positive gas ions to the solid, if this gradient indeed promotes their spontaneous loss. Furthermore, any gas species will diffuse away from the solid due to the presence of a chemical gradient. For both reasons, equilibrium does not describe sublimation.

Based on sublimation being an inherently disequilibrium process, the thermodynamic approach of comparing states is not an appropriate means for quantification. Foremost, the energies of formation do not describe the elastic energy stored in a solid. Second, the gas produced varies with the structure of the solid and its chemical bond type. Third, the speciation of the gas determined via mass spectrometry differs from the gas actually sublimated during typical experiments, since neutral species go undetected.

### 5.4. Implications

#### 5.4.1. The Energetics of Solids

We have shown that the vibrational energy in a solid plays no role in sublimation. Yet, the energetics of a solid has been depicted as vibrational (phonons), which is traceable to the long standing models of Debye and Einstein. These models are supported by comparison with heat capacity measurements. However, specific heat is defined by the response of the solid to heat added (Equation (8)), not to the heat stored. Stored heat depends on the constant of integration. Thus, *c_P_* and its closely related properties (per volume or per mole) describe augmentation of a solid’s energy, not the reservoir itself.

We have shown that the main energy reservoir of a solid is its rigidity. Young’s modulus, like density, is finite as *T* approaches 0 K (see, e.g., Figure 10 and Figure 12 in Ref. [5]). The constant of integration is thus ϒ at 0 K, which is reasonably approximated by ambient values (see [1] for figures and a summary of the literature).

Vibrations are activated thermally, so finite *T* is required for their existence. Possibly, the concept of zero point energy is a consequence of neglecting the elastic energy of a solid. This postulate is based on zero point energies of a gas being hypothetical, as all matter solidifies before this limit is reached.

Also relevant is the recognition that heat is generated during application of stress in the elastic regime [8]. Results of the present study, and our previous work [1,5] show that heat generation exists ubiquitously because vibrational collisions are partially inelastic and exist at all finite temperatures. Finite temperature describes all experimental determinations of elastic and other physical properties, so heat is also generated and lost from unstressed solids. So, stress is not the cause of heat emissions.

Ubiquitous heat loss, which is in accord with Stefan-Boltzmann’s law, goes unnoticed because the surroundings provide compensating heat that maintains the temperature of a body. Otherwise, experiments could not approach isothermal or adiabatic conditions. Also, the losses are rather small. Thus heat generation is not an essential component of the elastic description of solids.

#### 5.4.2. Possible Future Work

Several applications come to mind: Stability of a solid is related to its elastic energy reservoir. This has not been accounted for in accessing phase equilibria. Rather, the immeasurable quantity of entropy is utilized.

Rigidity of a solid impedes diffusion, not just on the surface, as discussed here, but also necessarily inside the body. Models of mass diffusion trace to the kinetic theory of gas, in which rigidity plays no part.

Use of self-consistent databases in thermodynamics (e.g., [60]) masks the flaws of omitting the main energy reservoir of solid. Although a useful framework for various calculations is provided, opportunity exists for misunderstanding. Perhaps the most revealing demonstration of the consequences of neglecting rigidity, is inference of mantle convection. Such models assume that the Earth behaves as a fluid, i.e., is infinitely deformable [61], when in fact, the hot, hydrostatic deep mantle is as rigid as Al_2_O_3_ at room temperature (see appendix tables in [13]). Natural convection is not possible, although forced movements can occur [62].

Regarding work of a fundamental nature, variations in the proportionality constant between Young’s modulus sublimation enthalpy in Equation (14) are of interest. The expectation of ⅓ is met in close packed metals (Section 4.5), but why ~⅕ is observed for many other structure-types (Table 1) needs further investigation, such as by computational methods.

Below ambient *T*, ϒ is constant, but decreases as *T* increases [63,64]. Decreasing Δ*H*_sub_ with *T* is suggested by data on refractory metals as a function of composition. The classical assumption of constant Δ*H*_sub_ needs reconsidering: i.e., experimental measurements of the dependence of Δ*H*_sub_ on temperature are needed for a better understanding.

## 6. Conclusions

Elasticity has been considered separately from thermodynamic assessment of solid behavior, with few exceptions [1,5]. From the 2nd law, heat added to matter performs work. Here we explore heat-generated work that removes constituents of the solid from its surface. The variety of materials examined provides insight into the process of sublimation.

Sublimation is a reaction. The reactant (parent solid) and the product (gas species) must be specified to quantify the energetics in a thermodynamic assessment. We have provided an independent means to ascertain the heat needed for sublimation at any given temperature from Young’s modulus, density, and molecular weight, namely Δ*H*_sub_ = ⅓ϒ *M*/*ρ*, which presumes that all bonds are broken. Both ϒ and *ρ* depend weakly on temperature, which explains the overall consistency in the thermodynamic database, despite its shortcomings. However, not all bonds are broken, particularly in non-metals and compounds, which explains disagreements among studies and divergent behavior of metals and insulators. The database on molecular solids is particularly impacted. By considering rigidity as the reservoir, we have ascertained the process of sublimation from available data on the products. Gas speciation greatly depends on the circumstances, which emphases that comparing states is a questionable means of probing sublimation.

## Figures and Tables

**Table 1:** Proportionality constants for the linear dependencies of sublimation enthalpy (Equation (14)) and thermal expansivity (Equation (11)) on Young’s modulus.

Type of Solid	Number *	Structure or Group	Sublimation Constant #2	Sublimated Gas Species ¶	Species Ref. ¶	Expansion Constant #1 §
metals	12	fcc (low to medium ϒ)	0.34			0.39
	16	hcp (low ϒ)	~0.36			0.34
	15	all bcc	polynomial †			~0.56
	4	bcc IA (very low ϒ)	0.68 ||			=1.1ϒ ^0.69^
	4	bcc VB (medium ϒ)	~0.41			-
	4	bcc VIB (high ϒ)	0.25	M^+^ ≥ M^−^	[27]	-
	5	IIA	0.21 ‡			-
other elements	4	diamond IVA	0.20 ‡	C_3_ ≥ C ≥ C_2_; some C^−^	[27,35]	~0.56
	10	VA+VIA+simple ices	0.21	clusters; H^+^ ≥ OH^−^, H_2_O	[31,32]	-
diatomic compounds	4	B1 XO	0.19	Ni+½O_2_; some NiO	[40]	~0.48
	11	B1 Alkali halides	0.23			~0.48
	3	B2 Cs halides	0.18			-
triatomic fluorides	5	fluorite XF_2_	0.16			-
IIB-VIA semiconductors	10	zinc blende and others	0.23	Si+Si_2_C+SiC_2_	[38]	-
molecular solids	5	diverse, complex	0.095	+ and ‚−ions; molecules	[29]	-
Equations used			$\frac{\rho \Delta H_{\mathrm{sub}}}{M}=\mathrm{const}.\Upsilon$			$\frac{\rho c_{P}}{\alpha_{\mathrm{vol}}}=\mathrm{const}.\Upsilon$

* Refers to the number of substances used in the fit. † Proportionality constant = 0.90 using *G*., instead of ϒ. || Lithium was not fit. ‡ Proportionality constant is largely controlled by the highest ϒ element (C, Be). ¶ Dominate species from mass spectrometry studies are shown with a reference. Charge states are listed where evidence is strong: see Section 2.3.2 for details. § Results from [1]: see Appendix A and Section 5 for discussion.

## Data Availability

The original contributions presented in this study are included in the article. Further inquiries can be directed to the author.

## Appendix A

Thermal expansion being opposed by rigidity of solids is briefly documented here. Figure A1a uses the same data (plus caesium) as in [1] (Figure 13 therein) which evaluated Equation (11) while neglecting the proportionality constant. Figure A1a differs from Ref. [1] by comparing different combinations of α, *ρ*, and ϒ. Figure A1b differs by distinguishing structure types and by adding data on simple cubic oxides from [51], to those used previously [1] (Figure 14a in this work) to test the geometrically estimated proportionality constant of [1]. Regarding the oxides, non-cubic Al_2_O_3_ controls the slope in Figure A1a.
